# Impacts of lung and tumor volumes on lung dosimetry for nonsmall cell lung cancer

**DOI:** 10.1002/acm2.12104

**Published:** 2017-06-28

**Authors:** Weijie Lei, Jing Jia, Ruifen Cao, Jing Song, Liqin Hu

**Affiliations:** ^1^ University of Science and Technology of China Hefei China; ^2^ Key Laboratory of Neutronics and Radiation Safety Institute of Nuclear Energy Safety Technology Chinese Academy of Sciences Hefei China; ^3^ Engineering Technology Research Center of Accurate Radiotherapy of Anhui Province Hefei China; ^4^ The Second Affiliated Hospital of Xuzhou Medical University Xuzhou China

**Keywords:** lung cancer, intensity‐modulated radiotherapy, volumetric‐modulated arc therapy

## Abstract

The purpose of this study was to determine the impacts of lung and tumor volumes on normal lung dosimetry in three‐dimensional conformal radiotherapy (3DCRT), step‐and‐shoot intensity‐modulated radiotherapy (ssIMRT), and single full‐arc volumetric‐modulated arc therapy (VMAT) in treatment of nonsmall cell lung cancers (NSCLC). All plans were designed to deliver a total dose of 66 Gy in 33 fractions to PTV for the 32 NSCLC patients with various total (bilateral) lung volumes, planning target volumes (PTVs), and PTV locations. The ratio of the lung volume (total lung volume excluding the PTV volume) to the PTV volume (LTR) was evaluated to represent the impacts in three steps. (a) The least squares method was used to fit mean lung doses (MLDs) to PTVs or LTRs with power‐law function in the population cohort (N = 32). (b) The population cohort was divided into three groups by LTRs based on first step and then by PTVs, respectively. The MLDs were compared among the three techniques in each LTR group (LG) and each PTV group (PG). (c) The power‐law correlation was tested by using the adaptive radiation therapy (ART) planning data of individual patients in the individual cohort (N = 4). Different curves of power‐law function with high R^2^ values were observed between averaged LTRs and averaged MLDs for 3DCRT, ssIMRT, and VMAT, respectively. In the individual cohort, high R^2^ values of fitting curves were also observed in individual patients in ART, although the trend was highly patient‐specific. There was a more obvious correlation between LTR and MLD than that between PTV and MLD.

## 1 INTRODUCTION

In radiotherapy (RT) of NSCLC, patients are at risk of radiation pneumonitis (RP) which is sometimes fatal.^1^ Patient characteristics, including tumor volume, shape, location, and lung volume often have a large range of variety, which increases the complexity and the level of challenging to decrease the lung dose.^2^, ^3^

In recent years, there has been a continuous increase in use of VMAT due to its rotational characteristic for much shorter treatment times and higher conformal dose distributions compared to ssIMRT and 3DCRT techniques. With this tendency, comparisons were performed among those techniques with debates.^4^, ^5^, ^6^, ^7^, ^8^ Current studies often only collected all volume of tumors into one group (normally with a large tumor volume range).^9^ Therefore, the detailed information could not be obtained on how the lung or tumor volume affected lung dosimetry in different techniques, which played an important role in pulmonary toxicity risk prediction.

In current RT, tumor volume is one of the commonly used patient parameters for decision of proper treatment modality in both conventional and unconventional fraction schemes. High tumor dose is normally inaccessible because of possible lung injury,^10^, ^11^, ^12^, ^13^, ^14^, ^15^, ^16^, ^17^ especially for large tumors. Discrepancy in lung volume among patients may cause differences in toxicity and potential of dose escalation for patients with approximate tumor volumes suggesting the need for an individualized treatment mode. But few studies have given their attention to these impacts in the current standard RT techniques. To investigate the effects of variations in normal lung or tumor volumes on normal lung dosimetry may benefit these clinical studies.

To our best knowledge, the impacts of lung and tumor volume variations on normal lung dosimetry in the above three techniques were still ambiguous, and it was necessary to find their correlation and a proper parameter to represent these impacts more accurate than tumor volume for its lack of attention to lung volume. In this study, MLD comparisons for patients with various characteristics of lungs and tumors (PTVs) were performed and LTR was proposed as the metric to represent the impacts.

## 2 METHODS

### 2.A Patient selection

After retrospectively reviewing the archives of lung cancer patients treated with RT combined chemotherapy at our department, 32 patients with stage I‐III NSCLC staged according to the IASLC 2009 were recruited in this study as the population cohort with various lung volumes, tumor volumes, and tumor locations. Permission to conduct the study was granted by the Research Ethics Board of the hospital. All simulations were performed with the Brilliance Big Bore CT 16‐slice scanner (Philips Medical Systems, Cleveland, OH, USA) with slices of a 5 mm thickness. All patients were in the supine position by using a fixation of a thermoplastic mask with their arms elevated with free‐breathing (FB). In the individual cohort, several patients previously treated with adaptive radiation therapy (ART) were selected as the individual cohort and each patient had four sets of images which were scanned prior treatment, at the end of week 2, 3, and 4 with FB, respectively.

### 2.B PTV and organs at risk delineation

To determine the clinical target volume (CTV), a margin of 8 mm around gross tumor volume (GTV) was used, including primary or metastatic lung tumor and involved mediastinum nodes (if visible in images). The PTV was created by adding an 8 mm uniform margin to CTV. A leaf margin of 2 mm was added to the PTV to improve the conformal index (CI) of PTV. Their localizations were classified according to their position on coronal sections at the centers of PTVs.

The PTV and the organs at risk (OAR), including ipsilateral lung, contralateral lung, spinal cord, heart, and esophagus, were outlined using lung window width and level settings (1600 HU,−600 HU) and mediastinal settings (400 HU, 20 HU) respectively followed by manually edits. The lung volume was defined as the volume of the total lung volume excluding the PTV.

In the case of inverse planning methods, a second volume was created and defined as the considered organ minus the PTV as an assistant area to avoid hot spots around PTV and to improve CI.

### 2.C Planning techniques: 3DCRT, ssIMRT, and VMAT

All plans were designed to deliver a prescription dose of 66 Gy in 33 fractions to PTV with XiO v4.6 TPS for 3DCRT, and the Monaco v3.2 TPS was used for ssIMRT and VMAT plans. All plans were delivered by the Elekta Axesse™ linear accelerator (Elekta, Crawley, United Kingdom) with the 160 MLC leaves of the Agility™ head using 6‐MV photons.

Once the treatment planning was completed, the plan was normalized to cover 95% of the PTV by the prescription dose. The dose volume constraints were set as follows: V20 < 30%, V30 < 20%, and MLD < 16 Gy. The maximum dose point for the spinal cord was 45 Gy. In addition, the plan optimization was also performed to keep the esophagus dose of V50 < 25% and mean esophageal dose <25 Gy, and the heart dose of V40 to 30%.

3DCRT planning was performed with the superposition dose calculation algorithm using 3–5 coplanar beams. Beam angles were configured to avoid unnecessary radiation to the contralateral lung and this depended on the individualized anatomic structure, PTV, and PTV location. And the collimator or wedge was used to optimize dose distribution if necessary.

The ssIMRT plans consisted of five coplanar beams, and beams were configured to cover the PTV with nonfixed angles. The VMAT plan consisted of one single full‐arc corresponded to a single 358° rotation, which started at the gantry angle of 179° and then counter‐clockwise rotated to stop at the gantry angle of 181°.

The dose volume constraints and relative priorities for both ssIMRT and VMAT were the same at the start of the optimization. The specific plan mode (ssIMRT or VMAT) was selected before optimization. The optimization was performed in two steps. The first step was performed by pencil beam dose calculation algorithm to obtain the optimal modulated fluence. During this process, objective parameters were adjusted to achieve optimal results until there was no gap between the goals which were previously adjusted and the results optimized. In the second step, the Monte Carlo dose calculation algorithm was used to optimize the segments aiming at small areas of targets. For ssIMRT, the minimum segment area was set to 2 cm^2^ and the minimum machine output per segment remained constant 4 MU. For VMAT, the maximum control points were set to 120 and the minimum segment width was 0.5 cm.

### 2.D Evaluation of LTRs

The evaluation was performed in three steps. Firstly, the least squares method was used to fit the MLDs to LTRs and PTVs with power‐law to test if there existed one correlation in the population cohort. To decrease the effects of individual characteristics (e.g., tumor location, subjectivity in planning) in one single plan, patients were divided into five groups according to the distribution of their PTVs or LTRs. Only the averaged values (LTRs, PTVs, and MLDs) in each group were used. Secondly, the effect of PTV variations on MLD was compared with that of LTR to investigate if there existed any difference or which one was more sensitive. Patients were divided into three groups by LTRs based on the fitting results of the first step and then by PTVs, respectively. MLDs were compared among the three techniques in the PGs and LGs, respectively. Thirdly, the power‐law correlation was fitted to test if this correlation was also existed in individual patients in adaptive radiation therapy (ART) by using their planning data.

### 2.E Statistics

All results were presented with mean value and standard deviation. MLDs were compared using the Wilcoxon signed‐rank test with *P* < 0.05 to be indicative of statistical significance for the groups in the population cohort with SPSS software version 19.0 (IBM, Chicago, USA). Spearman rank correlation was used to test the correlation between LTRs or PTVs and MLDs in the population cohort.

## 3 RESULTS

### 3.A Patient characteristics

The details of 32 patients were shown in Table 1. All the patients had no supraclavicular nodes and other concomitant lung diseases (e.g., emphysema). Their lung volumes varied from 1577 to 6123 ml and PTVs ranged from 16 to 375 ml with various locations.

**Table 1:** Patient and tumor statistical characteristics

Characteristic	N (Median)	%
Tumor stage		
III	15	47
II	11	34
I	6	19
Tumor location		
RUL	6	18
RML	5	16
RLL	7	22
LUL	9	28
LLL	5	16
PTV (ml)		
≤100	10 (66)	31
101–150	8 (118)	25
151–200	8 (160)	25
>200	6 (279)	19
Lung volume (ml)		
≤2500	7 (2264)	22
2501–3000	7 (2768)	22
3001–4000	11 (3667)	34
>4000	7 (4759)	22
LTR		
≤15	7 (13)	22
16–20	6 (18)	18
21–30	7 (25)	22
31–40	5 (32)	16
>40	7 (61)	22

LTR, ratio of the lung volume to planning target volume; PTV, planning target volume; RML, right middle lobe; LUL, left upper lobe; LLL, left lower lobe; RLL, right lower lobe; RUL, right upper lobe.

### 3.B Correlation in the population cohort

**Figure 1:**
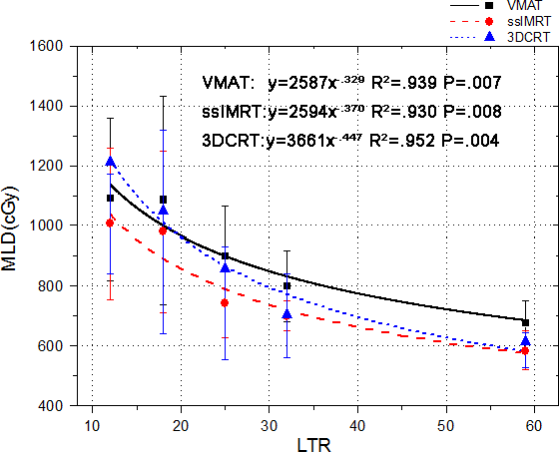
Fitted curves with error bars between averaged LTRs and averaged MLDs. For each curve, the five points (groups) represent the averaged values of 7, 6, 7, 5, and 7 patients in group 1 to group 5, respectively. LTR: ratio of lung volume to planning target volume. R^2^: the correlation coefficient.

All the 144 plans achieved the planning objectives. Spearman rank test showed a significant correlation between MLD and LTR (*P* = 0.001) or PTV (*P* = 0.044). Different curves with high R^2^ values were observed in Fig. 1 for 3DCRT, ssIMRT, and VMAT, respectively. The R^2^ values in PTV and MLD were much lower than those in LTR and MLD, and the correlation between PTV and MLD also had no statistically significance (PTV data not shown). The fitting results showed that there was a more obvious correlation between LTR and MLD than that between PTV and MLD.

For certain LTR, the large and different error bars in Fig. 1 showed that the effects of individual characteristics (especially PTV locations) on the three techniques were different. The upper limit values for VMAT were relatively larger compared with the other two techniques, and the lower limit values for 3DCRT were relatively smaller in nearly all the groups compared with VMAT. If the number of patients (still > = 5) in each group or the group number was changed (such as 4), there would be slight variations for the parameters of the power‐law correlation. But this has not changed the correlations between MLD and LTR with high R^2^ values for the three techniques.

### 3.C LTR and PTV

The MLD comparisons in PGs and LGs were shown in Table 2, which were divided according to the fitting results in the first step. At both sides of the LTR point of about 20 in Fig. 1, obvious difference of superiority in MLD could be observed between 3DCRT and VMAT. The LTRs around 20 point in the population cohort were divided into the middle‐LTR group, and the smaller and larger were divided into the small‐LTR group and large‐LTR group, respectively. To compare the relative sensitivity of LTR with PTV on MLD, the population cohort was accordingly divided into three groups (large‐PTV, middle‐PTV, and small‐PTV) by the PTVs. And the patient amounts in the corresponding groups were equivalent.

**Table 2:** Comparisons of MLDs in groups divided according to LTR and PTV

Subgroups	N (median)	VMAT	ssIMRT	3DCRT	VMAT vs ssIMRT	ssIMRT vs 3DCRT	3DCRT vs VMAT
		MLD (cGy), Mean ± SD			*P* value		
LTR							
≤15	7 (13)	1089 ± 270	1006 ± 252	1214 ± 166	**0.028**	**0.043**	0.091
16–25	10 (20)	991 ± 302	882 ± 251	981 ± 304	**0.007**	0.059	0.721
≥26	15 (36)	776 ± 153	657 ± 96	694 ± 130	**0.001**	0.211	**0.006**
PTV (ml)							
≥199	7 (263)	928 ± 318	867 ± 280	995 ± 331	**0.043**	0.237	0.499
123–188	10 (157)	1043 ± 266	930 ± 243	1023 ± 268	**0.013**	0.059	0.386
≤120	15 (87)	810 ± 209	687 ± 162	760 ± 253	**0.001**	**0.041**	0.088

PTV, planning target volume; LTR, ratio of lung volume to planning target volume; SD, standard deviation; MLD, mean lung dose.

The bold values indicated that they were statistically significant (*P* < 0.05).

In all the three PGs, MLDs for the ssIMRT were lower on average compared to VMAT (61 cGy, *P*=0.043; 113 cGy, *P* = 0.013; 123 cGy, *P* = 0.001) with statistically significance and 3DCRT without statistically significance except in the small‐PTV group (73 cGy, *P* = 0.041), and MLDs had no statistically significance for 3DCRT compared to VMAT. The comparison results in the three PGs were similar.

In all the three LGs, MLDs for the ssIMRT were also lower on average compared to VMAT (83 cGy, *P* = 0.028; 109 cGy, *P* = 0.007; 119 cGy, *P* = 0.001) with statistically significance. However, an obvious statistical difference was observed in the large‐LTR and small‐LTR groups compared to those in the corresponding groups in the PGs. MLDs for the 3DCRT was lower compared to VMAT (82 cGy, *P* = 0.006) in the large‐LTR group, and was higher compared to ssIMRT (208 cGy, *P* = 0.043) in the small‐LTR group with statistically significance. Additionally, for all the three techniques, MLDs in the large‐LTR and middle‐LTR groups were lower compared to the small‐PTV and middle‐PTV groups, respectively, and MLDs in the small‐LTR group were higher compared to the large‐PTV group. MLD was relatively more sensitive to LTR than to PTV.

Divisions of groups in the PGs and LGs were according to the fitting results. If the amount in the groups became a little larger or smaller, slight variations would be observed for the statistical result without changing the statistical significance. The results in Table 2 and Fig. 1 were in agreement with each other for LTRs and MLDs.

### 3.D Correlation in the individual cohort

**Figure 2:**
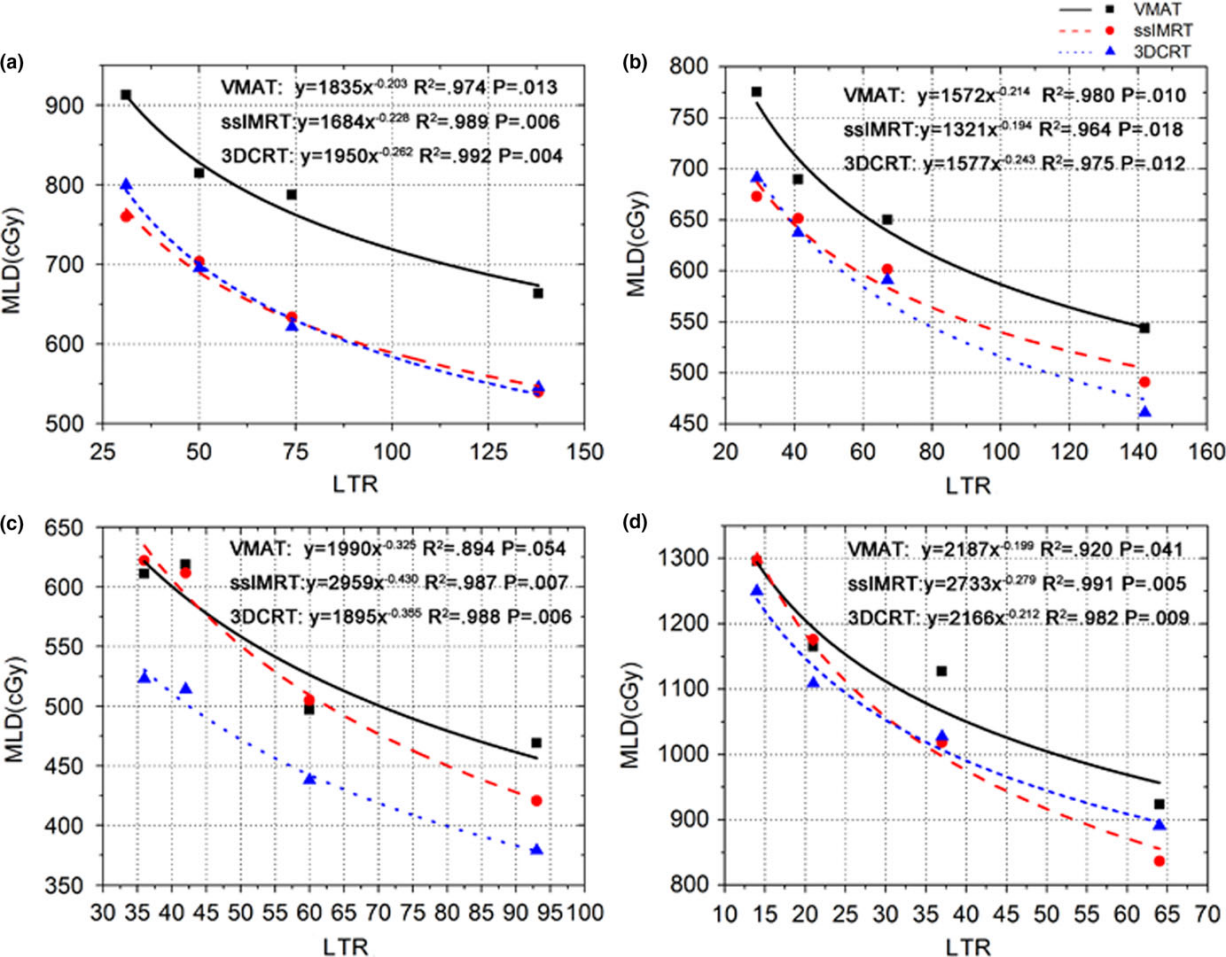
Fitted curves between LTR and MLD for individual patients with different tumor locations. Each point represents the changed LTR due to therapy at different treatment time. MLD: mean lung dose; LTR: ratio of lung volume to planning target volume. R^2^: the correlation coefficient.

For each patient in the individual cohort, 12 plans were designed in according to the image sets scanned in different treatment times for the three planning techniques. The fitting results were shown in Fig. 2. It was noticed that the correlations had high R^2^ values (with statistically significance), and the trends were different and highly patient‐specific at different LTR level for the three techniques.

As it was shown in Fig. 2, the correlation between MLD and LTR could be stated from the formula: MLD = aLTR^−b^, in which, parameters a and b were the coefficient and power exponent of the fitted curves, respectively.

## 4 DISCUSSION

Failure to decrease the RT‐induced lung injury is usually the main hurdle for an individual patient to acquire an optimal clinical outcome in RT of NSCLC. Multiple RT treatment techniques today provide the possible solutions to decrease the dose to the OARs. However, the variety of patient characteristics increases the difficulty to determine their impacts on lung dose in RT techniques. The current study investigated the impact of discrepancy in lung or tumor volumes on MLD in current standard RT techniques, and suggested one patient characteristic of LTR to represent this impact. The results indicated that the impact was obviously different and could not be neglected among the three compared techniques. There were different power‐law correlations between averaged LTR and averaged MLD for the three techniques.

Until recently, tumor volume was considered as one of the main patient characteristics in clinical treatment decision‐making. In a recent study by Wang et al. in 2013,^3^ the ratio of GTV to bilateral lung volume has been found to have a higher R^2^ value compared with tumor volume (GTV) in their correlations with MLD or V20 differences, in which the effect of different normal lung volume definition methods on lung DVH was investigated. In current study, we mainly concentrated on the discrepancy in lung and tumor volumes among patients, which might be meaningful to determine a proper treatment modality. Our data showed the limitations of using only tumor volumes to represent their impacts on MLDs in the three RT techniques. To improve the delivery of RT to avoid irradiating the normal lung, LTR might help the treatment planner to select the favorite treatment modality prior to the start of treatment planning. In our data, VMAT might not be the proper treatment modality compared with 3DCRT and ssIMRT for a patient with high LTR (e.g., ≥ 25), and 3DCRT might not be the favorite treatment modality for a patient with low LTR (e.g., < 15). It should be noticed that the above results were based on the population data. The different limit values of error bars in Fig. 1 indicated that the other individual characteristics, such as tumor locations, were also important factors which might have even larger impacts on MLDs for one certain technique. And the large differences of error bars between VMAT and 3DCRT also indicated that in some patients (e.g., peripheral PTV locations), to decrease the beam number might be helpful to decrease the MLD. Those individual effects could also be found in Fig. 2.

ART for lung cancer could achieve clinically relevant reductions in MLD with obvious reduction in tumor volume.^18^, ^19^, ^20^ In ART, plans were modified to be consistent with the tumor volume or shape variations due to treatment for a specific patient.^21^ In that case, the fitted curves would mainly be affected by tumor volume for similar tumor locations and lung volumes. And the correlations between MLD and tumor volume or LTR would be similar. To be objective for an actual diversity of lung tumors, completely different tumor locations were included in the individual cohort. Fitting curves in Fig. 2 showed a power‐law correlation between MLD and LTR with high R^2^ values, although the fitted curves were highly patient‐specific for being impacted by various individual characteristics. And in some patients, the difference in MLD might be relatively large at certain LTR level among the three techniques, which implied that much attention should be paid to LTR, tumor location and RT techniques when planning in ART.

Discrepancy in normal lung or tumor volume might also cause impacts on dosimetry of other OARs, including heart, esophagus, and cord. Nevertheless, these were not the primary objectives of this study. Patients in this study seldom involved those conditions in which their PTVs were immediately adjacent to critical structures (e.g., esophagus). In that case, arrangement of relatively fewer coplanar beams may be more difficult in sparing the OARs and improving therapeutic ratio. And in this study, we only generated plans of conventional dose fraction schemes. For SBRT, the volume characteristics and tumor localization will be more critical.^22^ Additional constraints for normal tissues need to be added for large dose fraction size, such as chest wall, esophagus, big blood vessels, and bronchial tree.^23^ These were not directly investigated in this study.

We recognize that our work is limited in several aspects. Firstly, the planning CT images were all conventionally acquired under FB conditions, and the variation of LTR due to respiratory motion was not considered. For a respiration‐correlated CT image set under FB, the lung volume varied between end‐expiration phase and end‐inspiration phase, which would cause slight effect on MLD during treatment. Secondly, we only had a limited number of patient cases in the population cohort and the subgroups, which might led to overestimation of reliability of the significant testing. And it was difficult to demonstrate the characteristic of tumor location on MLD with similar LTRs using limited data in our cohort, although the results have indicated that these effects might be relatively large on MLDs. And it is necessary to make further efforts using more cases. Thirdly, the ratio of the normal lung volume (excluding PTV) to PTV was used, and all our plans were designed to deliver a uniform dose to PTV. As some part of normal lung tissue was in CTV‐to‐PTV margins, it might be more proper to use GTV instead of PTV in the ratio which was actually correlated to tumor tissues. Nevertheless, that would not change the correlation between the ratio and MLD. Last but not least, the results were highly influenced by the planning techniques. The number and angle of beams in 3DCRT and ssIMRT were determined manually, which meant the experience of medical physicists had a great impact on results of the plans. Beam number and angle optimization^24^ may provide a slight improvement in MLD for the 3DCRT and ssIMRT plans. This effect, however, implied to support the result that 3DCRT and ssIMRT were preferred for patients with large‐LTRs.

## 5 CONCLUSION

The impacts of lung and tumor volumes were different on MLD among the three techniques. There were power‐law correlations between LTR and MLD in individual NSCLC patients who had replannings due to obvious reduction in tumor volume for the three planning techniques. To avoid irradiating the normal lung, 3DCRT and ssIMRT seemed to be preferred for a large‐LTR, and ssIMRT seemed to be preferred for a small‐LTR. The findings suggested that LTR was a useful patient characteristic and should be further evaluated in clinical investigations.

## ACKNOWLEDGMENTS

This work was supported by the National Natural Science Foundation of China (NSFC) (No. 11605233), the President of the Chinese Academy of Sciences Foundation for Young Spark Project (No. YZJJ201618), the Natural Science Foundation of Anhui Province (No. 1508085QH180), and the Industrialization Fund. In addition, the authors show their great appreciation to other members of the FDS Team for supports to this research.

## CONFLICT OF INTEREST

The authors declare no conflict of interest.
